# Skeletal muscle myosin heavy chain fragmentation following exercise may be linked to post‐exercise inflammation and remodelling

**DOI:** 10.1113/EP093340

**Published:** 2026-02-11

**Authors:** Dakota R. Tiede, Diego Bittencourt, J. Max Michel, Daniel L. Plotkin, Madison L. Mattingly, Derick A. Anglin, Christopher G. Vann, Zachary A. Graham, Timothy J. Broderick, Marcas M. Bamman, Michael D. Roberts

**Affiliations:** ^1^ School of Kinesiology, Nutrabolt Applied and Molecular Sciences Laboratory Auburn University Auburn Alabama USA; ^2^ Department of Physical Education, Laboratory of Neuromuscular Adaptations to Resistance Training Federal University University of São Carlos São Carlos Brazil; ^3^ Duke Molecular Physiology Institute Duke University Durham North Carolina USA; ^4^ Healthspan, Resilience, and Performance Research Florida Institute for Human and Machine Cognition Pensacola Florida USA; ^5^ Center for Exercise Medicine The University of Alabama at Birmingham Birmingham Alabama USA

**Keywords:** fragmentation, inflammation, myosin heavy chain, resistance training

## Abstract

The purpose of this exploratory investigation was to determine if acute post‐exercise skeletal muscle myosin heavy chain fragmentation (MyHC_frag_) coincides with alterations in molecular chaperones and proteolytic enzymes, select markers of mammalian target of rapamycin complex 1 (mTORC1) signalling, and/or specific gene expression signatures. Untrained males (*n* = 10, 23 ± 2 years) and females (*n* = 10, 23 ± 3 years) completed a bout of combined endurance and resistance exercise. Vastus lateralis muscle biopsies were taken before, 3 h and 24 h post‐exercise. Tissue was fractioned into myofibrillar (MF) and sarcoplasmic protein (SF) fractions for protein analysis. Differential RNA expression (DE) from those who experienced high and low MyHC_frag_ post‐exercise was also analysed via bulk RNA‐sequencing. MyHC_frag_ increased 24 h post‐exercise, albeit only four of 20 chaperone and proteolytic markers were concomitantly altered and none significantly correlated with 24 h post‐exercise MyHC_frag_. Given these null findings, we explored six participants who experienced the most post‐exercise MyHC_frag_ versus six who experienced the least MyHC_frag_ with the intent of examining if post‐exercise gene signatures or signalling differed. Although mTORC1 signalling markers were similar, 799 DE transcripts were identified 24 h post‐exercise. Pathway analysis on DE differences indicated that nine of the top 10 pathways enriched in high‐MyHC_frag_ participants were related to inflammation. High MyHC_frag_ participants also presented an upregulation in extracellular matrix remodelling genes at the 24 h post‐exercise time point. Though we lack immunohistochemical data, these findings suggest that post‐exercise MyHC_frag_ is associated with an upregulation in an inflammatory and remodelling signature, and longer‐term studies are needed to determine if these acute outcomes align with unique adaptive responses.

## Abstract (highlights)

- **What is the central question of this study?**
  Is myosin heavy chain fragmentation (MyHC_frag_) following resistance exercise in humans associated with a physiological signature in skeletal muscle?
- **What is the main finding and its importance?**
  Higher post‐exercise MyHC_frag_ is associated with a gene signature indicative of inflammation and remodelling. Future research is needed to determine if these acute outcomes translate to meaningful adaptive responses with chronic training.

## 1 INTRODUCTION

Skeletal muscle adaptation to resistance exercise involves a complex interplay of anabolic and catabolic processes that ultimately determine the adaptive response (Bamman et al., 2018; Roberts et al., 2023). While the anabolic responses to resistance exercise, including muscle protein synthesis (MPS) and hypertrophic signalling, have been extensively characterized, the role of exercise‐induced protein degradation remains less well understood. For instance, MPS can be directly measured using stable isotope tracers (e.g. deuterated water or ^13^C‐labeled amino acids) that get incorporated into newly synthesized proteins (Witard et al., 2022). However, assessing muscle protein breakdown (i.e. fractional breakdown rate, FBR) is more difficult to measure and often involves more invasive procedures including arteriovenous blood sampling combined with isotope infusions and blood flow assessments (Pasiakos & Carbone, 2014). Hence, surrogate markers of muscle proteolysis, such as mRNA and protein response or atrogene activity level assessments, are commonly assessed following either an acute exercise or chronic resistance training intervention, albeit there are variable responses in these outcomes reported in the literature (Godwin et al., 2023; Michel et al., 2025; Roberts et al., 2023).

Among the hundreds of skeletal muscle proteins comprising the contractile apparatus, myosin heavy chain (MyHC) constitutes approximately 40% of the total muscle protein pool and is central to force generation (Vann et al., 2020). We have previously demonstrated that skeletal muscle MyHC fragmentation (MyHC_frag_) can occur following acute resistance exercise (Plotkin et al., 2024). Using in vitro techniques, we also reported a significant relationship exists between MyHC_frag_ and calpain activity, suggesting that MyHC_frag_ may occur via calpain‐mediated proteolysis, similar to previous reports (Wette et al., 2021). However, while this may be a viable marker of post‐exercise proteolysis, its physiological significance is not known.

In the current study, we examined exercise‐induced MyHC_frag_ following a bout of combined endurance and resistance exercise. Given that heat shock proteins (HSPs) have been shown to interact with myosin isoforms in skeletal muscle (Ojima et al., 2018), we also assessed whether heightened post‐exercise MyHC_frag_ coincided with increased HSP expression or markers indicative of skeletal muscle proteolysis. Based on our previous research, we initially hypothesized that an elevation in MyHC_frag_ would occur following exercise, which would be accompanied by corresponding increases in proteolytic markers and HSPs. However, given that these initial findings did not yield fruitful insights, we reasoned that the physiological significance of MyHC_frag_ might be revealed through alternative molecular signatures. Thus, we performed an exploratory analysis comparing those who experienced the highest versus lowest post‐exercise MyHC_frag_ to determine if distinct gene expression patterns or anabolic signalling profiles differed between these two subgroups. We found that a high post‐exercise MyHC_frag_ response corresponds with an inflammatory and remodelling signature, and these findings as well as future directions are discussed herein.

## 2 METHODS

### 2.1 Ethical approval

The 20 individuals (10M/10F, 23.0 ± 2.6 years) included in this study were a subset of participants enrolled in a randomized clinical efficacy trial (McAdam et al., 2025). This study was conducted with prior review and approval from the University of Alabama at Birmingham (UAB) Institutional Review Board for Human Use (IRB reference number: F160512012) and in accordance with the *Declaration of Helsinki* (ClinicalTrials.gov Identifier: NCT03380923). Healthy college‐age males and females were recruited from the Greater Birmingham, Alabama area. Inclusion criteria required participants to be between the ages of 18 and 27 years old, deemed healthy by a health history questionnaire and medical history evaluation, and having not participated in regular exercise training for the past 12 months. Participants were ineligible for participation if they had a body mass index (BMI) ≥30 kg/m^2^, were currently pregnant or had a history of smoking. Verbal and written informed consent were obtained from eligible participants prior to enrolment in the study and participants consented to allow their biospecimens and data to be used in future research. Physiological and multi‐omic data have previously been published from these participants as part of larger analyses (Lavin et al., 2023, 2025; McAdam et al., 2025), with the characteristics of the subset used in this report presented in Table 1.

**TABLE 1:** Participant demographics.

Variable	Males (*n* = 10)	Females (*n* = 10)	Combined (*n* = 20)
Age (years)	23.3 ± 2.5	22.8 ± 2.5	23.0 ± 2.6
Height (m)	1.79 ± 0.06	1.69 ± 0.07	1.74 ± 0.08
Weight (kg)	80.82 ± 11.9	65.4 ± 9.7	73.5 ± 13.3
BMI (kg/m^2^)	25.2 ± 3.3	23.1 ± 4.1	24.2 ± 3.8
Lean mass (kg)	59.4 ± 8.7	41.4 ± 3.0	50.9 ± 11.1
Fat mass (kg)	19.0 ± 6.1	21.0 ± 7.3	19.9 ± 6.6

Data are presented as means ± standard deviation. Data provide descriptions of the participants analysed in this study.

### 2.2 Acute exercise bout

Participants were randomly assigned to complete an acute bout of traditional combined exercise (TRAD; *n* = 10) or a novel, militarily relevant high‐intensity combined exercise prescription (high intensity tactical training; HITT; *n* = 10). Before the acute exercise bout, participants completed four progressive familiarization sessions to: (i) ensure all exercises were performed safely and correctly; (ii) determine the target intensity for each exercise; and (iii) capture a molecular signature representative of an adaptive response rather than a response to overt damage. For more details regarding study protocols, see Lavin et al. (2025).

Participants in the TRAD group completed 30 min of steady‐state cycling on an ergometer at 70% of heart rate reserve. Following this, they completed three sets × ∼13 repetitions maximum (RM) of back squats, knee extensions, heel raises, chest press, overhead press, seated rows, wide grip pulldowns, triceps pushdowns and biceps curls with 60 s between sets. Additionally, three sets of bodyweight crunches were performed to accomplish as many reps as possible in 30 s. This resulted in 30 total sets being performed, in which a total of six sets loaded the vastus lateralis muscle, from which muscle biopsies were obtained.

The HITT group completed a 10‐round circuit consisting of the following exercises in order: cycling sprint no. 1, box jumps, kettlebell swings, burpees, battle ropes, wall balls, cycling sprint no. 2, dips, split squat jumps, and rowing sprint. Each exercise was performed with maximal effort for 30 s, followed by 30 s of rest before moving on to the next exercise. Following the circuit, the HITT group completed the same 10 resistance exercises as the TRAD group but in supersets and with intensity increased to target ∼9RM with only 30 s rest intervals between supersets.

Following the acute exercise bout, participants refrained from any further strenuous activity until after the 24 h post‐biopsy collection. Participants consumed a standardized supplement immediately post‐exercise containing 350 kcal (13 g of protein, 51 g of carbohydrate and 11 g of fat; Ensure Plus, Abbott Laboratories, Abbott Park, IL, USA) and then fasted until the 3 h post‐exercise biospecimen collections.

### 2.3 Tissue collection and processing

Vastus lateralis muscle biopsy samples were collected pre‐exercise, then 3 h and 24 h post‐exercise. Participants rested in a supine position on an exam table for ∼30 min before tissue collection. A percutaneous muscle biopsy was collected at each time point using a modified 5‐mm Bergstrom needle with suction. Portions (∼20–25 mg) of each sample were snap‐frozen in liquid nitrogen and stored in a −80°C freezer for protein and RNA analyses.

Muscle samples were crushed using a mortar and pestle in liquid nitrogen. Myofibrillar (MF) and sarcoplasmic protein (SF) fractions were isolated via the MIST method (Roberts et al., 2020). Briefly, ∼15 mg of crushed tissue was homogenized in MIST buffer 1 (25 mM Tris, pH 7.2, 0.5% Triton X‐100) using a tight‐fitting microtube pestle, then centrifuged at 1500 *g* for 10 min. The SF protein supernatant was pipetted off and placed into a new tube. The pellet was resuspended in MIST buffer 1 and centrifuged at 1500 *g* for 10 min as a wash step, and the supernatant was pipetted off until the pellet was apparently devoid of liquid. The pellet was then dried on ice before being resuspended in a MIST buffer 2 (20 mM Tris–HCl, pH 7.2, 100 mM KCl, 20% glycerol, 1 mM dithiothreitol (DTT), 50 mM spermidine). Tubes were then spun on a desktop centrifuge (1 min) and the MF fraction was transferred to a new tube. The soluble protein concentration for each fraction was determined using a commercially available BCA protein assay kit (Thermo Fisher Scientific, Waltham, MA, USA; cat. no. A55864) and spectrophotometer (Agilent Biotek Synergy H1 hybrid reader; Agilent, Santa Clara, CA, USA).

Calpain and 20S proteasome activities from MF and SF isolates were assayed using commercially available kits (Promega, Madison, WI, USA; cat. nos G8502 and G8622) according to the manufacturer's instructions and as we have previously reported (Michel et al., 2025, 2023; Scarpelli et al., 2024). Briefly, 50 µL of sample was diluted with diH_2_O at a 1:10 ratio and combined with either 50 mL of Calpain‐Glo or Proteasome‐Glo reagents and CaCl_2_ in black 96‐well plates. The plates were then shaken at 400 rpm for 30 s, incubated at room temperature for 15 min and read with a luminometer (gain = 135, read height = 7.00 mm, exposure = 20 s). Relative expression units were generated, and the values were normalized to the total protein loaded.

MF and SF isolates were also prepped for western blotting by diluting each sample with diH_2_O and 4× Laemmli buffer with added β‐mercaptoethanol (10% v:v) to a standard concentration (1 µg/µl). Samples were then heated for 5 min at 100°C and stored in a −80°C freezer until SDS‐PAGE. For electrophoresis, 5–15 µL of each sample was loaded onto a polyacrylamide gel (Bio‐Rad Laboratories, Hercules, CA, USA) and resolved using 180 V for ∼50 min. Proteins were then transferred onto a methanol‐activated polyvinylidene difluoride membrane (Bio‐Rad Laboratories) using 200 mA for 120 min. Membranes were stained with Ponceau dye for 10 min, washed with diH_2_O for ∼30 s, dried, and then imaged using the colorimetric setting (ChemiDoc Touch, Bio‐Rad; 0. 5 s exposure). Membranes were reactivated in methanol, blocked in 20 mL Tris‐buffered saline with Tween 20 (TBST) with 5% non‐fat bovine milk powder, and washed 3 × 5 min in TBST. Membranes were incubated in primary antibodies (1:200 for myosin heavy chain and 1:1000 v/v dilution for all other antibodies in TBST with 5% bovine serum albumin) overnight (48 h for phosphorylated targets) on a rocker at 4°C. Primary antibodies included: mouse anti‐MyHC (Developmental Studies Hybridoma Bank; Iowa City, IA, USA; cat. no.: A4.1025), rabbit anti‐αβ‐crystallin (CRYAB; Cell Signaling Technology, Danvers, MA, USA; cat. no.: 45844), rabbit anti‐HSP90 (Cell Signaling Technology, cat. no.: 4874), rabbit anti‐HSP70 (Cell Signaling Technology, cat. no.: 4872), rabbit anti‐20S proteasome core subunits (Enzo, Long Island, NY, USA; cat. no.: BML‐PW8155), rabbit anti‐calpain‐1 (Calp‐1; Cell Signaling Technology, cat. no.: 2556), rabbit anti‐calpain‐2 (Calp‐2; Cell Signaling Technology, cat. no.: 2556), rabbit anti‐ubiquitin (Cell Signaling Technology, cat. no.: 58395), rabbit anti‐p‐p70S6K (Thr389; Cell Signaling Technology, cat. no.: 34475), and rabbit anti‐p‐MKK3b (Ser189; Cell Signaling Technology, cat. no.: 9236). Following incubation with primary antibodies, membranes were washed 3 × 5 min in TBST and incubated for 1 h with horseradish peroxidase‐conjugated anti‐mouse or anti‐rabbit IgG (Cell Signaling Technology, cat. nos 7074 and 7076) diluted 1:2000 v/v in TBST. Membranes were washed once more for 3 × 5 min in TBST and then developed with chemiluminescent substrate (Millipore, Burlington, MA, USA). Membranes were digitally imaged using the chemiluminescent setting (ChemiDoc Touch, Bio‐Rad) for 5–60 s. For all proteins (including phospho targets) band densities were normalized to Ponceau densitometry values. Fold change values for proteolytic markers and chaperones were calculated by dividing each Ponceau‐normalized band density value by the aggregate PRE MF mean value for each target. Fold change values for phosphorylated targets were calculated by dividing each Ponceau‐normalized band density values by the aggregate PRE SF mean value for each target.

Detailed methods for RNA isolation, cDNA library preparation and RNA sequencing have been published previously (Lavin et al., 2023). In brief, RNA was isolated from ∼25 mg of snap frozen skeletal muscle using the Qiagen (Germantown, MD, USA) miRNeasy kit and a cooled Beadmill homogenizer. RNA quality was determined using an Agilent Bioanalyzer 2100, with the average RNA integrity number being ∼8.5. Total RNA was DNase‐treated, purified, then quantified again using Quant‐iT Ribogreen RNA assay and quality determined using an Agilent TapeStation 4200. Total RNA library synthesis was generated from 10 ng of RNA using the Takara Bio SMARTer Stranded Total RNA‐Seq Kit v2 Pico Input Mammal kit and sequenced at 101 × 9 × 9 × 101 using a NovaSeq 6000 to a depth of 80 million paired‐end reads. Sequenced data were aligned to GRCh38 using STAR with readcounts generated using featureCounts. FASTQ files and raw count matrices from this subset have been uploaded to Gene Expression Omnibus (GSE209880).

Raw data were imported into R (v4.4.3), where gene annotation was performed using the org.hs.db package. Low expressor transcripts were filtered based on mean and median library size thresholds, as described by Law et al. (2016). Data were normalized using trimmed mean m‐value (TMM) and analysed with quasi‐negative binomial regression via EdgeR. Genes were considered differentially expressed between the six highest and six lowest MyHC_frag_ respondents if *P *< 0.050, *Q* < 0.100, and fold‐change between respondent groups was >±1.5. Pathway analysis was conducted using PANTHER v19.0. Genes were used as the input for two separate PANTHER analyses (upregulated in high vs. low post‐exercise MyHC_frag_ groups and vice versa). Dot plots were created using ggplot2; the top 10 regulated pathways were selected for presentation herein.

### 2.4 Statistics

Group (TRAD vs. HITT) × time interactions were evaluated using a mixed‐effects model (restricted maximum likelihood) with subjects treated as a random effect to enable repeated measures analysis despite a missing data point of one participant. Given that there were no significant interactions between exercise protocols over time for MyHC_frag_ or other outcome variables, we present the main time effect data on the 20 pooled participants for all outcome variables. Tukey's HSD *post hoc* tests were conducted if a significant main effect was observed (*P *< 0.050). Pearson's correlations were performed to determine if the fold‐change in post‐exercise MyHC_frag_ was associated with changes in molecular chaperone or proteolytic markers in the 20 pooled participants. *R*‐values >±0.500 were considered moderate based on the framework established by Schober et al. (2018), albeit the threshold for significance was adjusted based on the number of correlations performed (i.e. *P *< 0.005 based on 10 comparisons being performed). For western blot analysis of low‐ versus high‐post‐exercise MyHC_frag_ groups, a two‐way repeated measures ANOVA was used, and Tukey's HSD *post hoc* test was conducted if a significant main effect or interaction was observed (*P *< 0.050). All data in tables and figures are presented as means ± standard deviation (SD). Aside from RNA data analyses described above, all other statistical analyses were conducted using Prism v.10 (GraphPad Software, Boston, MA, USA).

## 3 RESULTS

### 3.1 MyHC fragmentation

**FIGURE 1:**
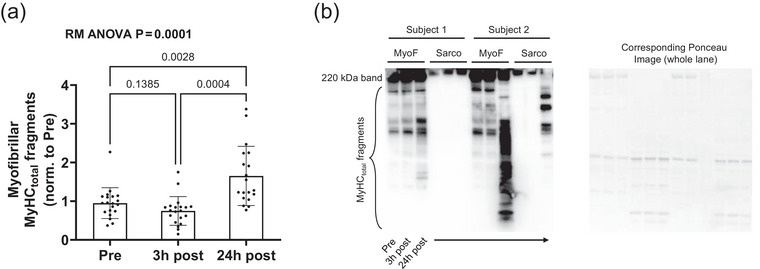
MyHC fragmentation response to exercise in all 20 participants. (a) MyHC fragmentation response in all 20 participants. Data are presented as bars (mean ± standard deviation values) with individual respondent data overlaid. (b) Representative western blot image for two subjects. Fold change values were calculated by dividing each Ponceau‐normalized band density value by the aggregate PRE MF mean value which was set to 1.00. RM ANOVA, repeated measures ANOVA; Pre, prior to exercise; 3 h post, 3 h post‐exercise; 24 h post, 24 h post‐exercise; MyoF, myofibrillar fraction; Sarco, sarcoplasmic fraction.

There was a significant main effect of time for MyHC_frag_ (Figure 1a) where MyHC_frag_ was significantly higher 24 h post‐exercise compared to Pre (*P* = 0.0028). Figure 1b shows a representative immunoblot of MyHC_frag_ in the MF and SF fractions in two participants.

### 3.2 Proteolytic markers

Table 2 contains the MF and SF proteolytic marker changes from Pre to 3 h and 24 h post‐exercise. Significant main effects of time were observed for SF Calp‐2 (Pre > 24 h post, *P* = 0.0045). When comparing these markers between fractions, the SF possessed significantly greater Calp‐1 protein (*P *< 0.0001), Calp‐2 protein (*P *< 0.0001), calpain activity (*P *< 0.0001), and 20S proteasome activity levels (*P *< 0.0001). Alternatively, the MF fraction possessed significantly greater 20S proteasome protein levels (*P *< 0.0001).

**TABLE 2:** Myofibrillar and sarcoplasmic proteolytic marker levels.

Marker	Pre	3 h post	24 h post	Time effect *P*‐value
MF calpain activity (RFU/µg protein) SF calpain activity^a^	700 (325) 1014 (299)	694 (431) 982 (308)	717 (407) 976 (685)	0.9358 0.9143
MF calpain‐1 protein (rel. expression) SF calpain‐1 protein^a^	1.00 (1.59) 17.0 (14.0)	0.45 (1.08) 17.4 (11.4)	0.29 (0.65) 12.1 (12.0)	0.1595 0.2651
MF calpain‐2 protein (rel. expression) SF calpain‐2 protein^a^	1.00 (1.13) 21.4 (6.7)	1.42 (1.97) 18.3 (5.5)	1.05 (1.35) 14.1 (6.6)^†^	0.1925 0.0016
MF 20S activity (RFU/µg protein) SF 20S activity^a^	66 (38) 120 (73)	63 (39) 131 (80)	57 (32) 98 (91)	0.6499 0.3521
MF 20S protein^a^ (rel. expression) SF 20S protein	1.00 (0.57) 0.00 (0.01)	0.82 (0.46) 0.09 (0.21)	1.01 (0.71) 0.02 (0.04)	0.4420 0.0923
MF poly‐Ub proteins (rel. expression) SF poly‐Ub proteins	1.00 (0.65) 1.02 (0.53)	0.91 (0.28) 1.19 (0.66)	1.09 (0.55) 1.00 (0.49)	0.4912 0.3001

Fold change values for proteolytic markers that were immunoblotted were calculated by dividing each Ponceau‐normalized band density value by the aggregate PRE MF mean value for each target which was set to 1.00. ^a^Higher values in one protein fraction versus another (significant *P*‐values indicated in text). †Downregulated versus Pre (significant *P*‐values indicated in‐text). MF, myofibrillar protein fraction; RFU, relative fluorescence unit; SF, sarcoplasmic protein fraction.

### 3.3 Heat shock proteins

Table 3 contains the MF and SF HSP changes from Pre‐to‐3 h and 24 h post‐exercise in all 20 participants. SF α,β‐crystallin (CRYAB) protein exhibited a significant change over time (Pre > 3 h post, *P* = 0.0269). An interesting and unanticipated finding was the presence of what appeared to be ∼140 kDa HSP70 bands predominantly localized to the SF. In consulting prior literature, it is apparent that HSP70 can form dimers in a context‐specific fashion and this is discussed more in later sections. It is also notable that SF HSP70 dimer levels exhibited significant changes over time (24 h post > Pre, *P* = 0.0120). When comparing these markers between fractions, the SF fraction possessed significantly greater chaperone protein levels for all assayed markers (CRYAB *P *< 0.0001, HSP90 *P *< 0.0001, HSP70 monomer *P *< 0.0001, HSP70 dimer *P *< 0.0001).

**TABLE 3:** Myofibrillar and sarcoplasmic chaperone protein levels.

Marker	Pre	3 h post	24 h post	Time effect *P*‐value
MF CRYAB protein (rel. expression) SF CRYAB protein^a^	1.00 (1.91) 9.60 (7.38)	1.97 (3.53) 4.88 (3.08)^†^	0.92 (1.65) 9.49 (8.57)	0.1023 0.0201
MF HSP90 protein (rel. expression) SF HSP90 protein^a^	1.00 (1.55) 5.66 (3.48)	1.37 (3.24) 4.73 (3.58)	1.25 (2.32) 5.71 (4.74)	0.7086 0.4258
MF HSP70 monomer (rel. expression) SF HSP70 monomer^a^	1.00 (0.81) 4.33 (2.64)	0.98 (1.01) 4.64 (2.70)	1.00 (0.88) 7.40 (7.85)	0.9891 0.1418
MF HSP70 dimer (rel. expression) SF HSP70 dimer^a^	1.00 (1.63) 31.1 (28.3)	1.05 (1.55) 35.5 (28.1)	1.22 (1.61) 57.0 (42.5)^*^	0.8673 0.0025

Fold change values were calculated by dividing each Ponceau‐normalized band density value by the aggregate PRE MF mean value for each target which was set to 1.00. ^a^Higher values in one protein fraction versus another (significant *P*‐values indicated in‐text). ^*^Upregulated versus Pre (significant *P*‐values indicated in‐text); †downregulated versus Pre (significant *P*‐values indicated in‐text). MF, myofibrillar protein fraction; SF, sarcoplasmic protein fraction.

### 3.4 Pearson's correlations between MyHC fragmentation and altered proteolytic and HSP markers

**FIGURE 2:**
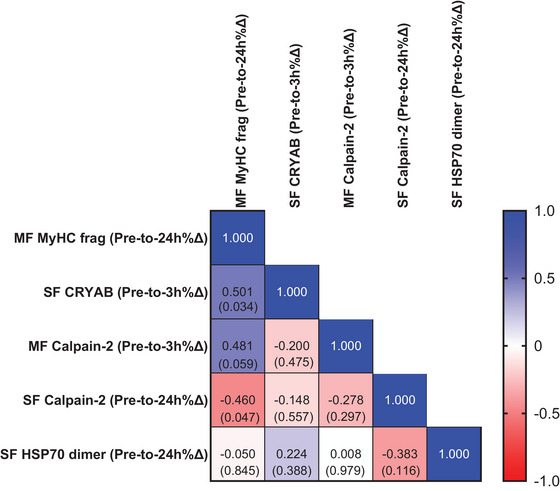
Select correlations between alterations in post‐exercise MyHC fragmentation and other assayed markers. Correlation *r*‐values (and associated *P*‐values in parentheses) between proteins that were altered post‐exercise versus 24 h post‐exercise MyHC fragmentation. Significance was adjusted to *P *< 0.005 based on 10 correlations being performed, and none of the associations reached this threshold. MF, myofibrillar protein fraction; SF, sarcoplasmic protein fraction. Protein abbreviations can be found in‐text.

**FIGURE 3:**
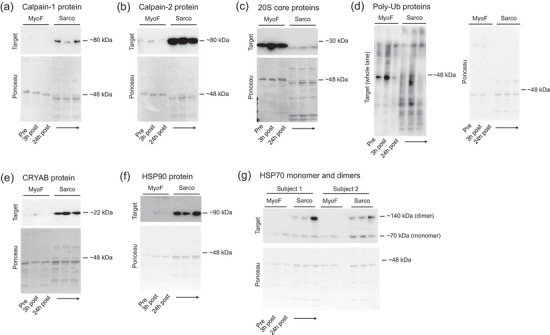
Representative western blot images. Legend: Images are representative western blots and corresponding Ponceau images for calpain‐1 protein (a), calpain‐2 protein (b), 20S core proteins (c), poly‐ubiquitinated proteins (d), CRYAB protein (e), HSP90 protein (f), and HSP70 monomer and dimers (g). Note: panel g shows two subjects and all other images show one subject. Pre, prior to exercise; 3 h post, 3 h post‐exercise; 24 h post, 24 h post‐exercise; MyoF, myofibrillar fraction; Sarco, sarcoplasmic fraction.

We next performed correlation tests on the 24 h post‐exercise percentage changes of MyHC_frag_ with 3 h or 24 h post‐exercise percentage changes in the proteolytic and HSP markers that were significantly altered in Tables 2 and 3 to determine if any significant associations existed (Figure 2). Only pre‐to‐3 h post‐exercise percentage changes in SF CRYAB (*r* = 0.501) showed a moderate correlation, albeit this did not reach statistical significance when adjusting for multiple comparisons (*P* = 0.0340). Representative images of all western blot targets can be found in Figure 3.

### 3.5 Gene expression and pathway analysis

**FIGURE 4:**
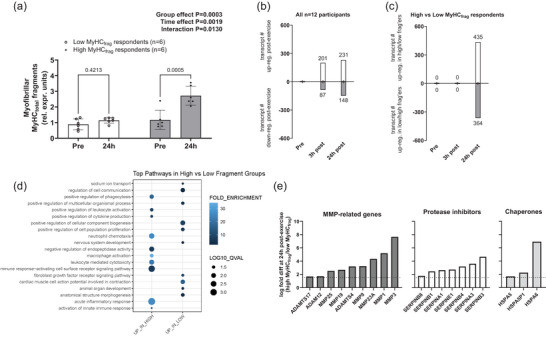
Gene expression profiling of low and high post‐exercise MyHC_frag_ responders. (a) Myosin heavy chain fragmentation (MyHC_frag_) data in six low and six high post‐exercise respondents showing a significant delineation; data are presented as bars (mean ± standard deviation values) with individual respondent data overlaid. (b) Bulk RNA‐seq in all 12 participants indicated 288 and 379 genes were dynamically altered 3 h and 24 h post exercise (±1.5 fold‐change vs. Pre and *Q* < 0.100). (c) Although no transcripts were differentially expressed between high versus low‐MyHC_frag_ respondents according to our significance thresholds, 799 genes were differentially expressed (DEGs) between respondent groups 24 h post‐exercise. (d) Predictive pathway analysis of these 24 h post‐exercise transcripts indicated cytokine signalling and inflammation persisted in high MyHC_frag_ participants, whereas this signature was absent in low MyHC_frag_ participants who instead presented an enrichment in transcripts related to remodelling and anabolism (e.g. cell proliferation, fibroblast growth factor signalling, cellular component biogenesis). (e) Of the 799 DEGs in panel (c), 19 genes related to extracellular matrix remodelling (e.g. MMPs), proteolysis (e.g. protease inhibitors) and chaperones were higher 24 h post‐exercise in high versus low MyHC_frag_ participants. Conversely, only select serine proteases (HGFAC, PCSK4, NAPSA, MASP1) and no MMP‐ or chaperone‐related genes were higher 24 h post‐exercise in low versus high MyHC_frag_ participants (data not shown).

To gain further insight into the relevance of post‐exercise MyHC_frag_, we performed a low versus high MyHC_frag_ analysis whereby participants were clustered into tertiles based on 24 h post‐exercise MyHC_frag_ values. In the highest tertile (*n* = 6, 5 males, 2 TRAD), MyHC_frag_ was elevated at 24 h post‐exercise compared to Pre (*P* = 0.0005, Figure 4a) compared to the lowest tertile at this time point (*P *< 0.0001). Conversely, in the lowest tertile (*n* = 6, two males, five TRAD), MyHC_frag_ was unaffected at 24 h versus Pre (*P* = 0.4213). These top and bottom tertiles were examined for differential gene expression patterns. Across these 12 participants a final count of 18,973 transcripts remained in the analysis following removal of low expressors and unannotated transcripts. No genes were differentially expressed between high‐ and low‐ MyHC_frag_ groups Pre (all *Q*‐values were >0.941) or 3 h post‐exercise (all *Q*‐values were >0.234; Figure 4b, c). However, robust differences were observed between these groups 24 h post‐exercise. Relative to low MyHC_frag_ participants, 364 transcripts were lower and 435 transcripts were higher in high MyHC_frag_ participants (>1.5‐fold change, *Q* < 0.100). A list of these differences can be found on an open access online data repository (https://doi.org/10.6084/m9.figshare.30213550.v1). The top 10 predicted Gene Ontology (GO) biological pathways using PantherDB.org are presented in Figure 4d. Notably, nine of the top 10 pathways predicted to be enriched in the high MyHC_frag_ group were associated with cytokine signalling and inflammation, while the top 10 pathways predicted to be enriched in the low MyHC_frag_ group were more closely aligned to canonical cell growth mechanics. The 435 transcripts that were higher 24 h post‐exercise in high versus low MyHC_frag_ participants were manually interrogated and 19 genes related to extracellular matrix remodelling (e.g. matrix metalloproteinase (MMPs) as well as ADAMST and SERPIN family genes) and chaperones were evident (Figure 4e). When examining the 364 transcripts that were lower in high versus low MyHC_frag_ participants 24 h post‐exercise, only select serine proteases (HGFAC, PCSK4, NAPSA, MASP1) and no MMP‐ or chaperone‐related genes were evident (data not shown).

### 3.6 Signalling responses in low‐ versus high‐MyHC_frag_ respondents

**FIGURE 5:**
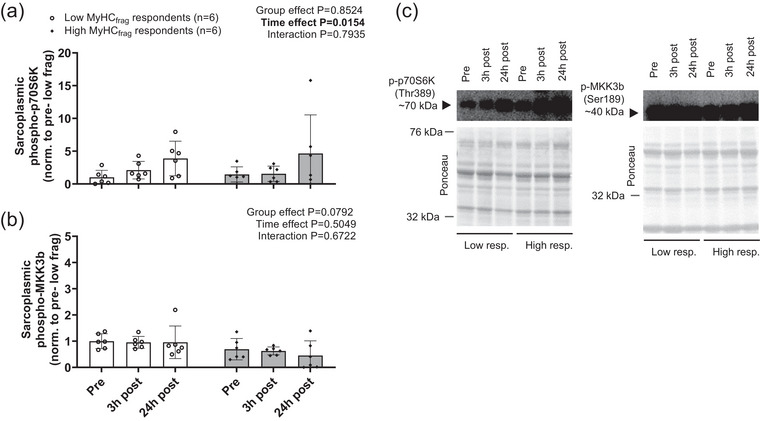
Select mTORC1 signalling responses between high and low MyHC_frag_ respondents. Legend: select mTORC1 signalling responses in the sarcoplasmic protein fraction of six low and six high post‐exercise MyHC_frag_ respondents including phosphorylated (p‐) p70S6K (a) and p‐MKK3b (b); data are presented as bars (mean ± standard deviation values) with individual respondent data overlaid. Panel c shows a representative western blot of the assayed markers. Other notes: Fold change values for phosphorylated markers were calculated by dividing each Ponceau‐normalized band density value by the aggregate PRE mean value for each target which was set to 1.00.

A significant main effect of time was observed for phosphorylated p70S6K protein levels (24 h post > Pre, *P* = 0.0171; Figure 5a). No significant effects or interaction were observed for p‐MKK3b protein levels (Figure 5b).

## 4 DISCUSSION

We sought to expand on our previous work to further characterize the physiological significance of MyHC_frag_ following an acute bout of exercise. Though post‐exercise MyHC_frag_ was not associated with changes in proteolytic proteins or molecular chaperones, exploratory transcriptomics on a subset of participants demonstrating the highest MyHC_frag_ suggests a coupling with post‐exercise inflammatory and tissue remodelling gene expression. We surmised that these differences may also influence key anabolic signalling markers; however, we observed no significant differences between high and low MyHC_frag_ groups in the post‐exercise anabolic proteins we investigated.

The most notable finding herein was that participants experiencing high post‐exercise MyHC_frag_ displayed a transcriptomic signature representative of a post‐exercise inflammatory response. This effect occurred despite performing four progressive familiarization sessions to reduce muscle damage via the repeated bout effect (Peake et al., 2005). The familiarization sessions progressed in both volume and intensity leading up to the experimental exercise bout, which was each participant's first exposure to the full volume and intensity. Research from Chen et al. (2010) suggests that four submaximal bouts of eccentric exercise confer similar protective effects as performing one maximal bout. Hubal et al. (2008) reported that the repeated bout effect was associated with increases in key mRNAs associated with a heightened post‐exercise inflammatory signature compared to the initial bout, suggesting that post‐exercise inflammatory responses may lead to beneficial longer‐term cellular adaptations. Hence, it is tempting to speculate that the increased inflammatory gene signature associated with high post‐exercise MyHC_frag_ may be a protective or adaptive mechanism, albeit more research is needed to confirm this hypothesis given that the current study was an acute interrogation. It is also notable that participants experiencing high post‐exercise MyHC_frag_ presented higher levels of genes mainly related to extracellular matrix remodelling at the 24 h post‐exercise time point (i.e. MMPs, as well as ADAMST and SERPIN family genes; Hjorth et al., 2015). These data are compelling given that they support that a more robust tissue remodelling response may occur in high post‐exercise MyHC_frag_ participants via enhanced MMP‐related gene expression. However, again, more chronic studies are needed to explore this hypothesis.

With our previous in vitro work, we observed that treatment of C2C12 myotubes with the potent calpain inhibitor calpeptin reduced MyHC_frag_ in both an anabolic and an atrophic model, suggesting the calpains play a role in inducing MyHC_frag_ (Plotkin et al., 2024). Despite this, in human *ex vivo* muscle tissue we observed no significant changes in calpain activity or Calp‐1 protein levels following acute exercise. Additionally, we observed a significant decrease in SF Calp‐2 protein levels 24 h post‐exercise, which was moderately, albeit not significantly, correlated with the MyHC_frag_ response. These data do not exclude the possibility that calpains may play a role in inducing MyHC_frag_, and our observations are likely due to biopsy sampling time points whereby some of these targets may have been altered greater than 24 h following the exercise session. Alternatively stated, MyHC_frag_ may precede the upregulation of proteolytic enzymes/activities, and the current analysis is limited in detecting changes that may occur days following the exercise bout. However, this hypothesis is again speculative, and time course studies are needed to determine if such a relationship exists.

HSPs serve several cellular functions including protection against cellular stressors such as hypoxia and ischaemia, regulating proteostasis via folding of nascent proteins, refolding of misfolded polypeptides, preventing formation of protein aggregates as well as directing misfolded proteins to be degraded via the ubiquitin proteasome system or autophagy (Acquarone et al., 2025; Liu & Steinacker, 2001; Noble et al., 2008). Additionally, HSPs may influence anabolic signalling cascades via interacting with mammalian target of rapamycin complex 1 (mTORC1) (Fennel et al., 2023). Though we anticipated that increased HSP expression would coincide with post‐exercise MyHC_frag_, we largely observed null effects. However, there was an increased presence of what appeared to be putative 140 kDa HSP70 dimers in the sarcoplasmic fraction at 3 h and 24 h post‐exercise (see Figure 3g). Several studies have examined the protein expression of HSP70 in skeletal muscle post‐exercise (de Lemos Muller et al., 2024; Paulsen et al., 2007; Thompson et al., 2002), but these studies have only examined the relative expression of the 70 kDa band. Hence, our observation of putative HSP70 dimers was unexpected given that no other human exercise studies have reported such findings in the past, and methodologically our reducing buffer and boiling prior to SDS‐PAGE would seemingly dissociate such dimers. Morgner et al. (2015) demonstrated that post‐translational modifications (particularly phosphorylation at T504 and multiple acetylation sites) substantially stabilize the HSP70 dimer interface through ionic interactions. Exercise is known to alter the phosphorylation and acetylation status of numerous muscle proteins, potentially creating a milieu for stable dimers. Moreover, exercise induces increased oxidative stress in skeletal muscle, and this catalyses an HSP response (Dimauro et al., 2016). Though speculative in the context of our data, increased oxidative stress may have promoted covalent cross‐linking of HSP70 through mechanisms such as disulfide bond formation (Hong et al., 2023). Although such covalent linkages would be resistant to SDS denaturation, we cannot exclude that the ∼140 kDa band represents HSP70 in complex with another protein of similar molecular mass. Notwithstanding, our findings are intriguing given that various in vitro models indicate the presence of 140 kDa HSP70 dimers in an ATP and temperature‐dependent fashion, such that the presence of ATP and increasing temperatures promote the formation of dimers (Angelidis et al., 1999; Trcka et al., 2019). It has also been reported that HSP70 dimers are needed to transfer certain proteins to other molecular chaperones (Morgner et al., 2015). The physiological relevance of potential post‐exercise HSP70 dimer formation, while interesting and relatively novel, was not fully examined herein and warrants future research consideration. Specifically, future human exercise studies employing immunoprecipitation followed by mass spectrometry are needed to definitively establish the identity of this band.

Whereas subjects with high post‐exercise MyHC_frag_ displayed significant upregulation in biological pathways related to the post‐exercise inflammatory response, those showing low post‐exercise MyHC_frag_ displayed a molecular signature that would indicate remodelling and cell anabolism. However, when examining changes in the phosphorylation status of proteins involved in anabolic signalling, no differences between groups were observed. We observed an increase in phosphorylation of p70S6K, which has been extensively shown to correlate with chronic changes in muscle mass (Roberts et al., 2023). Recently, it has been demonstrated that phosphorylation of MKK3b is highly correlated with the induction of myofibrillar protein synthesis (Zhu et al., 2025). However, despite a significant increase in p706SK phosphorylation, we did not observe significant changes in phosphorylation of MKK3b. In viewing these selective signalling markers, it appears that the magnitude of post‐exercise MyHC_frag_ does not affect post‐exercise anabolic signalling.

### 4.1 Experimental considerations

There are limitations with the current study. First, participants included in the study were untrained, otherwise healthy young adults. Individuals who have already adapted to resistance training may display an altered inflammatory response compared to untrained counterparts (Ihalainen et al., 2018). Second, given that this was a secondary analysis and there were tissue limitations, immunohistochemical assessments following exercise were not performed. This limitation is significant given that it would have been informative to examine potential associations between post‐exercise MyHC_frag_ and the presence of infiltrating immune cells (e.g. leukocytes, macrophages or neutrophils). Hence, a clear research direction moving forward is to establish if such a relationship exists. An additional consideration was our sample size, but the ability to collapse exercise doses and the repeated measures approach reduces this concern. With our exploratory omics analysis, however, we chose to divide the participants into tertiles based on the 24 h post‐exercise MyHC_frag_ response. This is indeed an unresolved limitation given the low sample size yielded, and it is notable that other strategies of determining responder status, such as *k*‐means clustering, may have generated different results. Finally, while we generated an observable signature at 24 h post‐exercise, these results cannot provide insight into the temporal response of MyHC_frag_ between the 3 h and 24 h time points as well as more extended post‐exercise timeframes.

### 4.2 Conclusions

In this relatively small cohort of familiarized but untrained young adults, we note substantial inter‐individual variation in the degree of post‐exercise MyHC_frag_. High fragmentation aligns with a transcriptional profile indicative of a muscle inflammatory response and extracellular matrix remodelling but is not coupled with post‐exercise changes in proteolytic signalling at the protein level, at least not at the time points analysed. Whether heightened MyHC_frag_ among a minority of familiarized participants is indicative of an advantage or disadvantage in long‐term training adaptations cannot be determined from this acute response study. Future research should investigate the MyHC_frag_ response to differing exercise intensities and should examine the relationship between MyHC_frag_, other inflammatory biomarkers (e.g. histological assessment of infiltrating immune cells) as well as indices of oxidative/mitochondrial stress, metabolic stress, membrane damage, or other indices of early‐phase muscle responses to combined endurance and resistance exercise.

## AUTHOR CONTRIBUTIONS

Dakota R. Tiede: conceived experiment, performed experiments, performed data analysis, constructed graphs, primarily drafted manuscript; Diego Bittencourt, J. Max Michel, Daniel L. Plotkin, Madison L. Mattingly, Derick A. Anglin, and Christopher G. Vann: assisted with experiments, provided edits to manuscript; J. Max Michel and Christopher G. Vann performed RNA‐sequencing data analysis; Zachary A. Graham: conceived experiment, provided edits to manuscript; Timothy J. Broderick: solicited funding, provided edits to manuscript; Marcas M. Bamman: conceived experiment, solicited funding for experimentation, provided edits to manuscript; Michael D. Roberts: conceived experiment, provided resources for experimentation, provided edits to manuscript.All authors have read and approved the final version of this manuscript and agree to be accountable for all aspects of the work in ensuring that questions related to the accuracy or integrity of any part of the work are appropriately investigated and resolved. All persons designated as authors qualify for authorship, and all those who qualify for authorship are listed.

## CONFLICT OF INTEREST

None declared.

## Data Availability

DEGs can be found at figshare.com (https://doi.org/10.6084/m9.figshare.30213550.v1) and other raw data can be obtained from the co‐corresponding author upon reasonable request (mdr0024@auburn.edu).

## References

[eph70217-bib-0001] Acquarone, D. , Bertero, A. , Brancaccio, M. , & Sorge, M. (2025). Chaperone proteins: The rising players in muscle atrophy. Journal of Cachexia, Sarcopenia and Muscle, 16(1), e13659. 39707668 10.1002/jcsm.13659 PMC11747685

[eph70217-bib-0002] Angelidis, C. E. , Lazaridis, I. , & Pagoulatos, G. N. (1999). Aggregation of hsp70 and hsc70 in vivo is distinct and temperature‐dependent and their chaperone function is directly related to non‐aggregated forms. European Journal of Biochemistry, 259(1–2), 505–512. 9914533 10.1046/j.1432-1327.1999.00078.x

[eph70217-bib-0003] Bamman, M. M. , Roberts, B. M. , & Adams, G. R. (2018). Molecular regulation of exercise‐induced muscle fiber hypertrophy. Cold Spring Harbor perspectives in medicine, 8(6), a029751. 28490543 10.1101/cshperspect.a029751 PMC5983156

[eph70217-bib-0004] Chen, T. C. , Chen, H. L. , Lin, M. J. , Wu, C. J. , & Nosaka, K. (2010). Potent protective effect conferred by four bouts of low‐intensity eccentric exercise. Medicine and Science in Sports and Exercise, 42(5), 1004–1012. 19997007 10.1249/MSS.0b013e3181c0a818

[eph70217-bib-0005] de Lemos Muller, C. H. , Schroeder, H. T. , Farinha, J. B. , Lopez, P. , Reischak‐Oliveira, A. , Pinto, R. S. , de Bittencourt Junior, P. I. H. , & Krause, M. (2024). Effects of resistance training on heat shock response (HSR), HSP70 expression, oxidative stress, inflammation, and metabolism in middle‐aged people. Journal of Physiology and Biochemistry, 80, 161–173. 37930617 10.1007/s13105-023-00994-w

[eph70217-bib-0006] Dimauro, I. , Mercatelli, N. , & Caporossi, D. (2016). Exercise‐induced ROS in heat shock proteins response. Free Radical Biology and Medicine, 98, 46–55. 27021964 10.1016/j.freeradbiomed.2016.03.028

[eph70217-bib-0007] Fennel, Z. J. , Ducharme, J. B. , Berkemeier, Q. N. , Specht, J. W. , McKenna, Z. J. , Simpson, S. E. , Nava, R. C. , Escobar, K. A. , Hafen, P. S. , Deyhle, M. R. , Amorim, F. T. , & Mermier, C. M. (2023). Effect of heat stress on heat shock protein expression and hypertrophy‐related signaling in the skeletal muscle of trained individuals. American Journal of Physiology‐Regulatory, Integrative and Comparative Physiology, 325(6), R735–R749. 37842742 10.1152/ajpregu.00031.2023

[eph70217-bib-0008] Godwin, J. S. , Telles, G. D. , Vechin, F. C. , Conceicao, M. S. , Ugrinowitsch, C. , Roberts, M. D. , & Libardi, C. A. (2023). Time course of proteolysis biomarker responses to resistance, high‐intensity interval, and concurrent exercise bouts. Journal of Strength and Conditioning Research, 37(12), 2326–2332. 37506190 10.1519/JSC.0000000000004550

[eph70217-bib-0009] Hjorth, M. , Norheim, F. , Meen, A. J. , Pourteymour, S. , Lee, S. , Holen, T. , Jensen, J. , Birkeland, K. I. , Martinov, V. N. , Langleite, T. M. , Eckardt, K. , Drevon, C. A. , & Kolset, S. O. (2015). The effect of acute and long‐term physical activity on extracellular matrix and serglycin in human skeletal muscle. Physiological Reports, 3(8), e12473. 26290530 10.14814/phy2.12473 PMC4562559

[eph70217-bib-0010] Hong, Z. , Gong, W. , Yang, J. , Li, S. , Liu, Z. , Perrett, S. , & Zhang, H. (2023). Exploration of the cysteine reactivity of human inducible Hsp70 and cognate Hsc70. Journal of Biological Chemistry, 299(1), 102723. 36410435 10.1016/j.jbc.2022.102723 PMC9800336

[eph70217-bib-0011] Hubal, M. J. , Chen, T. C. , Thompson, P. D. , & Clarkson, P. M. (2008). Inflammatory gene changes associated with the repeated‐bout effect. American Journal of Physiology‐Regulatory, Integrative and Comparative Physiology, 294(5), R1628–R1637. 18353886 10.1152/ajpregu.00853.2007

[eph70217-bib-0012] Ihalainen, J. K. , Schumann, M. , Eklund, D. , Hamalainen, M. , Moilanen, E. , Paulsen, G. , Hakkinen, K. , & Mero, A. A. (2018). Combined aerobic and resistance training decreases inflammation markers in healthy men. Scandinavian Journal of Medicine & Science in Sports, 28(1), 40–47. 28453868 10.1111/sms.12906

[eph70217-bib-0013] Lavin, K. M. , Graham, Z. A. , McAdam, J. S. , O'Bryan, S. M. , Drummer, D. , Bell, M. B. , Kelley, C. J. , Lixandrao, M. E. , Peoples, B. , Tuggle, S. C. , Seay, R. S. , Van Keuren‐Jensen, K. , Huentelman, M. J. , Pirrotte, P. , Reiman, R. , Alsop, E. , Hutchins, E. , Antone, J. , Bonfitto, A. , …, Bamman, M. M. (2023). Dynamic transcriptomic responses to divergent acute exercise stimuli in young adults. Physiological Genomics, 55(4), 194–212. 36939205 10.1152/physiolgenomics.00144.2022 PMC10110731

[eph70217-bib-0014] Lavin, K. M. , O'Bryan, S. M. , Pathak, K. V. , Garcia‐Mansfield, K. , Graham, Z. A. , McAdam, J. S. , Drummer, D. J. , Bell, M. B. , Kelley, C. J. , Lixandrao, M. E. , Peoples, B. , Seay, R. S. , Torres, A. R. , Reiman, R. , Alsop, E. , Hutchins, E. , Bonfitto, A. , Antone, J. , Palade, J. , …, & Bamman, M. M. (2025). Divergent multiomic acute exercise responses reveal the impact of sex as a biological variable. Physiological Genomics, 57(5), 321–342. 40014011 10.1152/physiolgenomics.00055.2024

[eph70217-bib-0015] Law, C. W. , Alhamdoosh, M. , Su, S. , Dong, X. , Tian, L. , Smyth, G. K. , & Ritchie, M. E. (2016). RNA‐seq analysis is easy as 1‐2‐3 with limma, Glimma and edgeR. F1000Res, 5, 1408. 10.12688/f1000research.9005.1 PMC4937821 27441086

[eph70217-bib-0016] Liu, Y. , & Steinacker, J. M. (2001). Changes in skeletal muscle heat shock proteins: Pathological significance. Frontiers in Bioscience, 6, D12–25. 11145923 10.2741/liu

[eph70217-bib-0017] McAdam, J. S. , Craig, M. P. , Graham, Z. A. , Peoples, B. , Tuggle, S. C. , Seay, R. S. , Lavin, K. M. , Gargus, A. B. , O'Bryan, S. M. , Yang, S. , Drummer, D. J. , Kelley, C. J. , Peri, K. , Bell, M. B. , Aban, I. , Cutter, G. R. , Mahyari, A. , Wen, Y. , Zhang, J. , …, Bamman, M. M. (2025). Multidimensional biocircuitry of exercise adaptation: Integrating in vivo and ex vivo phenomics with miRNA mapping. Physiological Genomics, 57(9), 526–550. 40658616 10.1152/physiolgenomics.00068.2025

[eph70217-bib-0018] Michel, J. M. , Godwin, J. S. , Plotkin, D. L. , McIntosh, M. C. , Mattingly, M. L. , Agostinelli, P. J. , Mueller, B. J. , Anglin, D. A. , Kontos, N. J. , Berry, A. C. , Vega, M. M. , Pipkin, A. A. , Stock, M. S. , Graham, Z. A. , Baweja, H. S. , Mobley, C. B. , Bamman, M. M. , & Roberts, M. D. (2025). Effects of leg immobilization and recovery resistance training on skeletal muscle‐molecular markers in previously resistance‐trained versus untrained adults. Journal of Applied Physiology, 138(2), 450–467. 39819075 10.1152/japplphysiol.00837.2024

[eph70217-bib-0019] Michel, J. M. , Godwin, J. S. , Plotkin, D. L. , Mesquita, P. H. C. , McIntosh, M. C. , Ruple, B. A. , Libardi, C. A. , Mobley, C. B. , Kavazis, A. N. , & Roberts, M. D. (2023). Proteolytic markers associated with a gain and loss of leg muscle mass with resistance training followed by high‐intensity interval training. Experimental Physiology, 108(10), 1268–1281. 37589512 10.1113/EP091286 PMC10543615

[eph70217-bib-0020] Morgner, N. , Schmidt, C. , Beilsten‐Edmands, V. , Ebong, I. O. , Patel, N. A. , Clerico, E. M. , Kirschke, E. , Daturpalli, S. , Jackson, S. E. , Agard, D. , & Robinson, C. V. (2015). Hsp70 forms antiparallel dimers stabilized by post‐translational modifications to position clients for transfer to Hsp90. Cell Reports, 11(5), 759–769. 25921532 10.1016/j.celrep.2015.03.063 PMC4431665

[eph70217-bib-0021] Noble, E. G. , Milne, K. J. , & Melling, C. W. (2008). Heat shock proteins and exercise: A primer. Applied Physiology, Nutrition and Metabolism, 33(5), 1050–1075. 10.1139/H08-069 18923583

[eph70217-bib-0022] Ojima, K. , Ichimura, E. , Suzuki, T. , Oe, M. , Muroya, S. , & Nishimura, T. (2018). HSP90 modulates the myosin replacement rate in myofibrils. American Journal of Physiology‐Cell Physiology, 315(1), C104–C114. 29561661 10.1152/ajpcell.00245.2017

[eph70217-bib-0023] Pasiakos, S. M. , & Carbone, J. W. (2014). Assessment of skeletal muscle proteolysis and the regulatory response to nutrition and exercise. Iubmb Life, 66(7), 478–484. 25052691 10.1002/iub.1291

[eph70217-bib-0024] Paulsen, G. , Vissing, K. , Kalhovde, J. M. , Ugelstad, I. , Bayer, M. L. , Kadi, F. , Schjerling, P. , Hallen, J. , & Raastad, T. (2007). Maximal eccentric exercise induces a rapid accumulation of small heat shock proteins on myofibrils and a delayed HSP70 response in humans. American Journal of Physiology‐Regulatory, Integrative and Comparative Physiology, 293(2), R844–R853. 17522120 10.1152/ajpregu.00677.2006

[eph70217-bib-0025] Peake, J. , Nosaka, K. , & Suzuki, K. (2005). Characterization of inflammatory responses to eccentric exercise in humans. Exercise Immunology Review, 11, 64–85. 16385845

[eph70217-bib-0026] Plotkin, D. L. , Mattingly, M. L. , Anglin, D. A. , Michel, J. M. , Godwin, J. S. , McIntosh, M. C. , Kontos, N. J. , Bergamasco, J. G. A. , Scarpelli, M. C. , Angleri, V. , Taylor, L. W. , Willoughby, D. S. , Mobley, C. B. , Kavazis, A. N. , Ugrinowitsch, C. , Libardi, C. A. , & Roberts, M. D. (2024). Skeletal muscle myosin heavy chain fragmentation as a potential marker of protein degradation in response to resistance training and disuse atrophy. Experimental Physiology, 109(10), 1739–1754. 39180757 10.1113/EP092093 PMC11442757

[eph70217-bib-0027] Roberts, M. D. , McCarthy, J. J. , Hornberger, T. A. , Phillips, S. M. , Mackey, A. L. , Nader, G. A. , Boppart, M. D. , Kavazis, A. N. , Reidy, P. T. , Ogasawara, R. , Libardi, C. A. , Ugrinowitsch, C. , Booth, F. W. , & Esser, K. A. (2023). Mechanisms of mechanical overload‐induced skeletal muscle hypertrophy: Current understanding and future directions. Physiological Reviews, 103(4), 2679–2757. 37382939 10.1152/physrev.00039.2022 PMC10625844

[eph70217-bib-0028] Roberts, M. D. , Young, K. C. , Fox, C. D. , Vann, C. G. , Roberson, P. A. , Osburn, S. C. , Moore, J. H. , Mumford, P. W. , Romero, M. A. , Beck, D. T. , Haun, C. T. , Badisa, V. L. D. , Mwashote, B. M. , Ibeanusi, V. , & Kavazis, A. N. (2020). An optimized procedure for isolation of rodent and human skeletal muscle sarcoplasmic and myofibrillar proteins. Journal of Biological Methods, 7(1), e127. 32201709 10.14440/jbm.2020.307 PMC7081056

[eph70217-bib-0029] Scarpelli, M. C. , Bergamasco, J. G. A. , Godwin, J. S. , Mesquita, P. H. C. , Chaves, T. S. , Silva, D. G. , Bittencourt, D. , Dias, N. F. , Medalha Junior, R. A. , Carello Filho, P. C. , Angleri, V. , Costa, L. A. R. , Kavazis, A. N. , Ugrinowitsch, C. , Roberts, M. D. , & Libardi, C. A. (2024). Resistance training‐induced changes in muscle proteolysis and extracellular matrix remodeling biomarkers in the untrained and trained states. European Journal of Applied Physiology, 124(9), 2749–2762. 38653795 10.1007/s00421-024-05484-5

[eph70217-bib-0030] Schober, P. , Boer, C. , & Schwarte, L. A. (2018). Correlation coefficients: Appropriate use and interpretation. Anesthesia & Analgesia, 126(5), 1763–1768. 29481436 10.1213/ANE.0000000000002864

[eph70217-bib-0031] Thompson, H. S. , Clarkson, P. M. , & Scordilis, S. P. (2002). The repeated bout effect and heat shock proteins: Intramuscular HSP27 and HSP70 expression following two bouts of eccentric exercise in humans. Acta Physiologica Scandinavica, 174(1), 47–56. 11851596 10.1046/j.1365-201x.2002.00922.x

[eph70217-bib-0032] Trcka, F. , Durech, M. , Vankova, P. , Chmelik, J. , Martinkova, V. , Hausner, J. , Kadek, A. , Marcoux, J. , Klumpler, T. , Vojtesek, B. , Muller, P. , & Man, P. (2019). Human stress‐inducible Hsp70 has a high propensity to form ATP‐dependent antiparallel dimers that are differentially regulated by cochaperone binding. Molecular & Cellular Proteomics, 18(2), 320–337. 30459217 10.1074/mcp.RA118.001044 PMC6356074

[eph70217-bib-0033] Vann, C. G. , Roberson, P. A. , Osburn, S. C. , Mumford, P. W. , Romero, M. A. , Fox, C. D. , Moore, J. H. , Haun, C. T. , Beck, D. T. , Moon, J. R. , Kavazis, A. N. , Young, K. C. , Badisa, V. L. D. , Mwashote, B. M. , Ibeanusi, V. , Singh, R. K. , & Roberts, M. D. (2020). Skeletal muscle myofibrillar protein abundance is higher in resistance‐trained men, and aging in the absence of training may have an opposite effect. Sports (Basel), 8(1), 7. 31936810 10.3390/sports8010007 PMC7022975

[eph70217-bib-0034] Wette, S. G. , Birch, N. P. , Soop, M. , Zugel, M. , Murphy, R. M. , Lamb, G. D. , & Smith, H. K. (2021). Expression of titin‐linked putative mechanosensing proteins in skeletal muscle after power resistance exercise in resistance‐trained men. Journal of Applied Physiology, 130(3), 545–561. 33356984 10.1152/japplphysiol.00711.2020

[eph70217-bib-0035] Witard, O. C. , Bannock, L. , & Tipton, K. D. (2022). Making sense of muscle protein synthesis: A focus on muscle growth during resistance training. International Journal of Sport Nutrition and Exercise Metabolism, 32(1), 49–61. 34697259 10.1123/ijsnem.2021-0139

[eph70217-bib-0036] Zhu, W. G. , Thomas, A. C. Q. , Wilson, G. M. , McGlory, C. , Hibbert, J. E. , Flynn, C. G. , Sayed, R. K. A. , Paez, H. G. , Meinhold, M. , Jorgenson, K. W. , You, J. S. , Steinert, N. D. , Lin, K. H. , MacInnis, M. J. , Coon, J. J. , Phillips, S. M. , & Hornberger, T. A. (2025). Identification of a resistance‐exercise‐specific signalling pathway that drives skeletal muscle growth. Nature Metabolism, 7(7), 1404–1423. 10.1038/s42255-025-01298-7 PMC12875287 40374925

